# Orthostatic Intolerance in Older Persons: Etiology and Countermeasures

**DOI:** 10.3389/fphys.2017.00803

**Published:** 2017-11-09

**Authors:** Nandu Goswami, Andrew P. Blaber, Helmut Hinghofer-Szalkay, Jean-Pierre Montani

**Affiliations:** ^1^Gravitational Physiology and Medicine Research Unit, Institute of Physiology, Medical University of Graz, Graz, Austria; ^2^Department of Biomedical Physiology and Kinesiology, Simon Fraser University, Burnaby, BC, Canada; ^3^Department of Medicine/Physiology, University of Fribourg, Fribourg, Switzerland

**Keywords:** syncope, exercise, mental arithmetic, water drinking, aging, falls

## Abstract

Orthostatic challenge produced by upright posture may lead to syncope if the cardiovascular system is unable to maintain adequate brain perfusion. This review outlines orthostatic intolerance related to the aging process, long-term bedrest confinement, drugs, and disease. Aging-associated illness or injury due to falls often leads to hospitalization. Older patients spend up to 83% of hospital admission lying in bed and thus the consequences of bedrest confinement such as physiological deconditioning, functional decline, and orthostatic intolerance represent a central challenge in the care of the vulnerable older population. This review examines current scientific knowledge regarding orthostatic intolerance and how it comes about and provides a framework for understanding of (patho-) physiological concepts of cardiovascular (in-) stability in ambulatory and bedrest confined senior citizens as well as in individuals with disease conditions [e.g., orthostatic intolerance in patients with diabetes mellitus, multiple sclerosis, Parkinson's, spinal cord injury (SCI)] or those on multiple medications (polypharmacy). Understanding these aspects, along with cardio-postural interactions, is particularly important as blood pressure destabilization leading to orthostatic intolerance affects 3–4% of the general population, and in 4 out of 10 cases the exact cause remains elusive. Reviewed also are countermeasures to orthostatic intolerance such as exercise, water drinking, mental arithmetic, cognitive training, and respiration training in SCI patients. We speculate that optimally applied countermeasures such as mental challenge maintain sympathetic activity, and improve venous return, stroke volume, and consequently, blood pressure during upright standing. Finally, this paper emphasizes the importance of an active life style in old age and why early re-mobilization following bedrest confinement or bedrest is crucial in preventing orthostatic intolerance, falls and falls-related injuries in older persons.

## Introduction

In most persons, the hemodynamic and neurovascular responses to orthostatic challenge produced by standing up are adequate to stabilize arterial blood pressure and to maintain cerebral blood flow after standing. However, numerous patients come to clinics with the complaint of dizziness upon standing (Rapp et al., 2012). Syncope, defined as a transient self-limiting loss of consciousness, is the end-point of cardiovascular stability. Clinical symptoms preceding syncope can include: nausea, blurring of vision, dizziness, and a sudden decrease in mean arterial pressure (MAP; Grasser et al., 2009a,b) due to a reduction in stroke volume, heart rate, or peripheral resistance. These responses elicited by upright posture characterize the condition of orthostatic intolerance (OI).

The etiology of cardiovascular instability that leads to orthostatic intolerance is multifactorial (Custaud et al., 2002; Weimer and Williams, 2003). Among several causes, orthostatic intolerance can arise due to alterations in cerebral blood flow (Blaber et al., 2011) and/or the control of cardiovascular regulation. Etiology of cardiovascular dysregulation contributing to OI could be centrally located (e.g., arising due to changes in cardiac stretch receptors) or peripherally located (e.g., arising due to limitations in increasing peripheral vascular resistance, venoconstriction and/or sympathetic activity loss, or functional changes in arterial baroreflex).

### Aging, orthostatic intolerance, and countermeasures

There is a steep global trend toward the demographic aging of populations. It is projected that people aged 65+ years in the EU will almost double over the next 50 years, reaching up to 151 million in 2060 (URL1) leading to increasing public health costs as well as costs for older people and their families. The negative consequences of aging such as illness or injury often require admission to hospital. However, the bedrest confinement that occurs during hospitalization is considered a major factor in physiological deconditioning and functional decline and can contribute to a downhill spiral of increasing risk of frailty, and orthostatic intolerance, leading to falls (Mühlberg and Sieber, 2004). A systematic review by Heinrich et al. (2010) indicates that 0.85–1.5% of all health care costs are dedicated solely to the consequences of falls. Therefore, understanding the mechanisms that contribute to falls as well as how risk of falls and falls-related injuries can be reduced is an important aspect in geriatric healthcare delivery (Blain et al., 2016; Bousquet et al., 2017; Broadbent et al., 2017).

As more than 80% of the time during hospital admission is spent lying in bed in older persons (Pedersen et al., 2013), bedrest confinement during hospitalization and its negative effects pose particular challenges in the care of the senior citizens in the acute and chronic care setting. Prolonged periods of physical inactivity or bedrest are associated with negative metabolic and functional effects (Agostini et al., 2010; Pisot et al., 2016; Soavi et al., 2016). Some of these include deconditioning in the cardiovascular, skeletal and neuromuscular systems as well as potential deficits in brain function and structure (Grogorieva and Kozlovskaia, 1987; Leblanc et al., 1990; Traon et al., 1998; Perhonen et al., 2001; Pisot et al., 2008; Lipnicki and Gunga, 2009; Rittweger et al., 2009; Dolenc and Petric, 2013; Marusic et al., 2014, 2016; Li et al., 2015; Cassady et al., 2016).

Further complicating this situation is the fact that orthostatic intolerance incidence is markedly accelerated with intravascular instrumentation (Stevens, 1966) or with increased heat stress (Crandall, 2000), and is greater in taller persons (Ludwig and Convertino, 1994) and in anxiety states (Smith et al., 1994).

In addition to reviewing the state of the art knowledge regarding orthostatic intolerance, this review also examines specific countermeasures that can be used against orthostatic intolerance. For instance, orthostatic tolerance has been shown to be improved by exercise (Howden et al., 2004), water drinking (Schroeder et al., 2002), and mental arithmetic (Goswami et al., 2012a). Other countermeasures that could potentially prevent or mitigate orthostatic intolerance can include simple measures such as standing up slowly (de Bruïne et al., 2017) and/or compression of abdominal region (Figueroa et al., 2010). For bed confined persons, countermeasures can include cognitive training (Goswami et al., 2015) and nutritional supplementation (Muscaritoli et al., 2017)—with and without physical activity—in mitigating orthostatic intolerance.

## Orthostatic challenge and orthostatic intolerance

Orthostatic challenge can lead to problems such as dizziness when the cardiovascular response to this challenge is inadequate. In the upright (orthostatic) position, the hemodynamic responses may not be enough to sustain the arterial blood pressure, and syncope may ensue. In a healthy person, upon standing up, 10–15% (~700 mL) of blood volume can pool due to gravitational effects in the legs (Naschitz and Rosner, 2007), with 50% of this shift occurring within the first 20–30 s (Toska and Walloe, 2002). The increased hydrostatic pressure induced by standing leads to: increases in transmural pressure in lower extremities; fluid movement from the vascular compartment to the interstitial space; and a decrease in plasma volume (Hinghofer-Szalkay and Greenleaf, 1987). This could potentially cause reductions in venous return, cardiac pre-load and cardiac output. In persons with normal cardiovascular regulatory capability, however, mean arterial blood pressure is maintained by compensatory increases in heart rate and total peripheral resistance (Rowell, 1993).

The autonomic nervous responses to standing are based on a balance between parasympathetic and sympathetic activity: the reflex tachycardia indicates vagal withdrawal and involves increases in sympathetic activity (Ramirez-Marrero et al., 2007). Hormonal responses, in comparison to autonomic neurogenic reflexes, play only a minor role in the early, and rapid hemodynamic responses during standing up.

### Age-related changes in orthostatic challenge induced responses

It is known that older persons are particularly prone to falls. The increase in falls due to postural hypotension and loss of postural stability is of major concern with aging. Age-associated changes in baroreflexes may also play a role in older subjects: advancing age is associated with increasing barosensory vessel wall stiffness and decreasing cardio-vagal autonomic control effectiveness (Monahan et al., 2001). There are additional important factors outlined below which can lead to orthostatic intolerance in older persons. However, before discussing these aspects it is necessary to outline the cardiovascular responses—as well the role skeletal muscles play in enhancing venous return—that occur during postural changes from supine to upright.

#### Cardiovascular responses to standing upright

Standing up decreases aortic and carotid blood pressure thus leading to unloading of the aortic arch and carotid sinus baroreceptors. This results in a rapid increase of heart rate via vagal withdrawal and slower elevation of heart rate and peripheral vascular resistance through sympathetic activation to ensure that arterial blood pressure is maintained (Rowell, 1993). During standing, the baroreflex-mediated sympathetic activity has minimal effect on venous tone in the lower limbs due to scarce sympathetic innervation in the veins within limb muscles (Fuxe and Sedvall, 1965; Samueloff et al., 1966). As a consequence, quiet standing results in extensive venous pooling in the legs due to absence of the action of the skeletal (muscle) pump. Contraction of lower limb muscles (along with one-way venous valves) pumps the pooled blood in the veins back to the heart, increases cardiac pre-load, and consequently cardiac output (Guyton et al., 1962; Ten Harkel et al., 1994).

Musculoskeletal activities of the lower limbs during standing have primarily been studied in relation to the central postural control system which generates proper balance corrections in the upright stance by coordinating sensory-motor control components (somatosensory, visual ocular, vestibular) to naturally optimize body positioning (Dichgans and Diener, 1989). However, the resultant slight postural sway during standing also serves as an important contributor in promoting venous return (Inamura et al., 1996; Murata et al., 2012; Blaber et al., 2013). Surprisingly, despite it being long known that the skeletal muscle pump helps in the maintenance of blood pressure (Guyton et al., 1962), cardiovascular and postural reflexes have been investigated as independent control mechanisms (Winter, 1995; Fadel, 2008). In addition, the influence of the skeletal muscle on the cardiovascular hemodynamic responses have been considered to be mechanical rather than reflex-mediated (Guyton et al., 1962; Ten Harkel et al., 1994).

### Postural instability with aging

Postural disturbances, which can arise due to external or internal causes in daily life, can lead to problems with balance during standing. Standing is maintained by a fine balance between the ability to detect postural disturbances and the generation of proper responses. However, these abilities worsen as age increases, and in older persons can cause imbalance and greater falls risk (Blaszczyk et al., 1994; MacKey and Robinovitch, 2006; Hsiao-Wecksler and Robinovitch, 2007). In addition, aging is associated with worsening of the functioning of the somatosensory and motor systems, which in turn can lead to poor standing balance (Lord et al., 1991; Hurley et al., 1998).

The alteration in standing balance is not only a problem of older persons. Changes in reactive postural responses have been seen in healthy young persons with reductions in somatosensory perception in laboratory experiments as well as in patients with peripheral neuropathy or alterations in vestibular function. An understanding of how the cardiovascular, postural and skeletal muscle systems interact to maintain upright posture is important in the development of treatment and training strategies for individuals for whom one or both systems are compromised.

#### The cardio-postural model

As introduced above, the control relationships between muskulo-skeletal, postural and cardiovacular systems is important and should be investigated. Studies conducted by Claydon and Hainsworth (2006) showed a link between postural sway and prevention of syncope. They reported that participants who had poor tilt table orthostatic tolerance but never fainted during normal standing showed greater postural sway than patients who experienced frequent syncopal episodes (Claydon and Hainsworth, 2006). In addition, Novak et al. (2007) proposed a cardio-locomotion coupling conceptual model in which muscle traction forces generated during walking pump venous blood and propel it to the heart with a step synchronized rhythm. These observations demonstrate the role of the skeletal (muscle) pump in cardiovascular control in conditions of insufficient vascular control.

**Figure 1:**
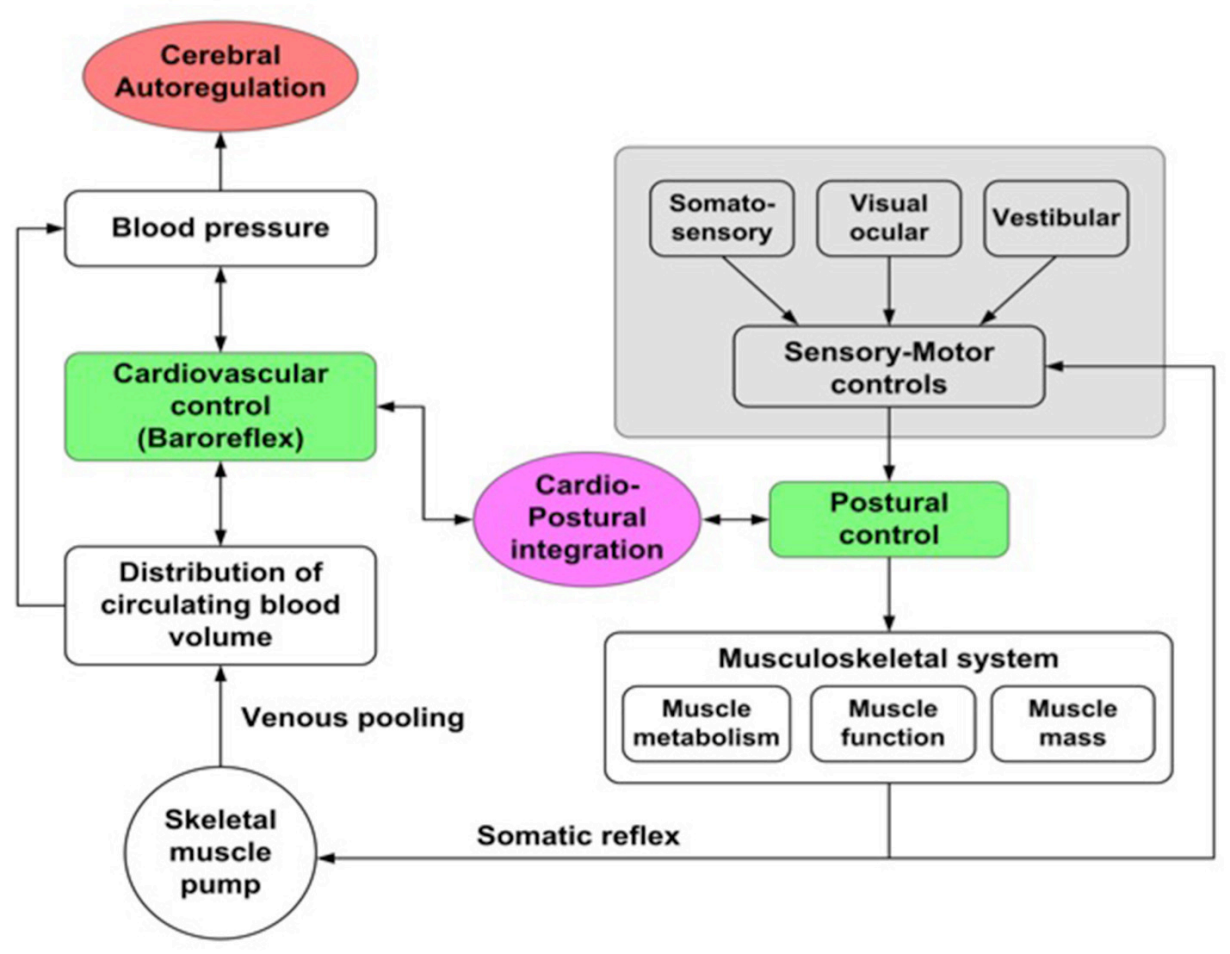
Cardio-postural interactions. Illustration of the cardio-postural components associated with mechanisms that prevent falls: **Left**—cardiovascular component of the regulation of blood pressure (blood volume, heart rate and vascular resistance); **Right**—sensory motor input components related to postural control; **Center**—cardio-postural integration: hypothesized as baroreflex activation of skeletal muscle pump.

Based on some studies in which a correlation between postural sway and blood pressure (BP) was observed, a new physiological model—*cardio-postural interactions*—which includes the interactions between cardiovascular control and postural changes has been developed (Souvestre et al., 2008; Blaber et al., 2009; Goswami et al., 2012b; Figure 1). Using this model, a relationship between calf muscle activation, measured with electromyography (EMG) and blood pressure during passive standing has been observed: during passive standing increased EMG activity was associated with subsequent increases in BP and decreased EMG activity followed by decreases in BP (Souvestre et al., 2008; Blaber et al., 2009). Additionally, Garg et al. (2014a) have also reported that older individuals have significant alterations in the relationship between BP and EMG activity. These investigators observed that in non-fainting older subjects the cardio-postural relationship was similar to young subjects; however, the percentage of time of significant coherence between EMG and BP variation increased with decreasing frequency. In the young age group, a similar frequency-based difference was not observed, which could indicate a cardio-postural behavior shift toward larger time scales (i.e., lower frequencies) with increasing age (Garg et al., 2014b). Verma et al. (2017) also showed that causal influence of skeletal muscle pump activity on blood pressure was significantly reduced with aging. Further understanding of the degree and mode of interaction between the cardiovascular control and centrally-regulated sensory-motor controls and how cardio-postural interactions are affected by bedrest confinement as well as by disease states is required.

Recently, Rodriguez et al. (2017) reported that there is no difference in spontaneous baroreflex sensitivity between stroke patients and healthy controls during standing up from a sitting posture. However, the gain values from SBP to EMG impulse were attenuated in patients, which suggests a post-stroke impairment of muscle-pump and baroreflex. This correlates with the finding that the BP drop upon standing in stroke patients approached the criteria for OH (orthostatic hypotension) (see later sections) and that the stroke patients showed a slightly longer BP recovery time. The impairment of muscle pump and baroreflex could arise due to muscle atrophy after bedrest in patients with stroke and/or by affecting nerve pathways to the muscular system. Further research into recording and analyzing motor unit activation and recruitment should be carried out.

### Orthostatic hypotension (OH) and aging

Orthostatic hypotension is common in older adults. The prevalence of asymptomatic orthostatic hypotension [which is defined as a reduction in diastolic BP ≥ 10 mmHg or systolic BP ≥ 20 mmHg, with BP measured supine and at 3-min standing] in 5,201 randomly selected persons aged 65 and older with natural history of—or with risk factors for—cerebrovascular or cardiovascular diseases was found to be 16.2% (Rutan et al., 1992). This prevalence increased to 18.2% when subjects in whom the sit to stand test was aborted due to dizziness upon standing were included (Rutan et al., 1992). Based on different studies, the prevalence of OH varies from 5 to 50% among elderly subjects (reviewed in Weiss et al., 2004).

It has been reported that OH is significantly associated with problems in walking and/ or falls, and histories of transient ischemic attacks and even myocardial infarction (Rutan et al., 1992). Indeed, OH is one of the main risk factors for falls (Tinetti et al., 1988). In older persons, falls are among the top five causes of death (Kannus et al., 2005).

As blood pressure is generally known to be lower in summer than in winter, the question whether seasonal variations in blood pressure affect OH incidence needs also to be further examined. Weiss et al. (2006) studied OH variation between seasons in 502 older male and female in-patients (mean age 81.6 years). Each older person performed orthostatic tests, 30 min after meals, three times per day. In their study, OH was documented in 35% of the population. While the researchers did not observe baseline BP differences between seasons, in older persons the drop in blood pressure upon standing up in the morning was greater in summer. Furthermore, orthostatic hypotension was more common in summer vs. winter months (38 vs. 27%; *p* = 0.02), and after taking into account all the confounders, orthostatic hypotension risk was found to be 64% higher in summer (Weiss et al., 2006).

### Orthostatic hypotension vs. intermediate BP drop

Due to the limited physiological reserve, older persons are more susceptible to changes in environment or pathological states. Hence, it is not surprising that some older patients complain of light-headedness/dizziness when standing up even though their blood pressure does not fall into the OH range. Intermediate BP drop, defined as reduction of 5–9 mmHg in diastolic BP and/or 10–19 mmHg in systolic BP, can act as an OH predictor. Intermediate BP drop is also associated with higher mortality rates (discussed in Weiss et al., 2004). The current Joint National Committee (JNC) recommendations suggest that a 10 mmHg BP reduction during orthostatic loading—when associated with symptoms—should be considered as clinically relevant (see Weiss et al., 2004).

### Postprandial hypotension and aging

Aging is accompanied by an increase in the tendency for blood pressure to decrease following a meal. Classically, hypotension following a meal (postprandial hypotension) is the arbitrary decrease in systolic BP ≥ 20 mmHg or postprandial reduction in systolic BP to <90 mmHg within 2 h following a meal (Jansen and Lipsitz, 1995). The maximal decrease in BP occurs usually between 30 and 60 min post-meal, but it may occur later in some individuals (Luciano et al., 2010). This decrease in BP is associated with a number of pathological events, including greater falls incidence (Le Couteur et al., 2003), syncope due to failure to maintain compensatory tachycardia and normal noradrenaline levels (Lipsitz et al., 1986), coronary events, stroke, and overall mortality (Aronow and Ahn, 1997).

The constitution of a meal seems to contribute toward postprandial hypotension. Carbohydrates (e.g., glucose) show blood pressure reducing effect (Potter et al., 1989). Indeed, older persons with no signs and symptoms of postprandial hypotension show blood pressure decreases following ingestion of a meal rich in carbohydrates (Lipsitz and Fullerton, 1986). Meal ingestion leads to blood pooling in the abdominal region/ vasculature but blood pressure is maintained due to the appropriate homeostatic responses (Lipsitz et al., 1993). In patients with dysautonomia, however, meal ingestion may be accompanied with a decrease in peripheral resistance and reductions in blood pressure. This could potentially lead to the development of presyncopal signs, symptoms or even orthostatic intolerance (Heseltine et al., 1990; Lipsitz et al., 1993).

Post-prandial hypotension has been reported in a large proportion of healthy individuals (Jones et al., 1998; Vloet et al., 2005; Van Orshoven et al., 2010). Additional risk factors for postprandial hypotension include specific co-morbid conditions, such as non-insulin-dependent diabetes mellitus (Jones et al., 1998), especially if accompanied by some degree of autonomic neuropathy (Sasaki et al., 1992), autonomic dysfunction (Shannon et al., 2002), hypertension (Grodzicki et al., 1998), Alzheimer's disease (Idiaquez et al., 1997), and Parkinson's disease (Loew et al., 1995). In addition, older patients with polypharmacy, especially those who use diuretics and psychotropic medications, are more likely to be affected by hypotension following a meal ingestion (Luciano et al., 2010).

Postprandial hypotension is often under-recognized among older persons (Luciano et al., 2010). In a study evaluating 85 older persons (mean age 80 ± 7 years) admitted to geriatric departments in Dutch hospitals, 67% presented a significant post-meal decrease in SBP of 34 ± 4 mmHg. Interestingly, orthostatic hypotension accompanied 52% of patients with an average systolic blood pressure decrease of 44 ± 4 mmHg following standing, and 37% of patients had both orthostatic and postprandial hypotension (Vloet et al., 2005). Indeed, a prospective study in 499 nursing home dwellers (>62 years, mean age 80) showed that the maximal decrease in systolic BP after a meal was a risk factor for syncope, falls, new coronary events or new stroke, and contributed significantly to greater mortality (Aronow and Ahn, 1997). In a study of 179 randomly selected low-level-care older residents (65 years and older, mean age 83 years) in long-term health facilities, postprandial hypotension was shown to be a powerful predictor of deaths arising due to all causes (Fisher et al., 2005).

Post-meal drop in blood pressure is also concomitant with asymptomatic cerebrovascular damage in patients with essential hypertension (Kohara et al., 1999). This observation was extended to residents of the general community (65 ± 9 years old, *n* = 1308), who were free from coronary heart disease or heart failure and participating in a general health checkup. A higher prevalence of cerebral lacunar infarctions as evidenced by MRI was shown in subjects with postprandial hypotension compared with controls (Tabara et al., 2014).

### Effects of skin temperature changes on orthostatic intolerance

Temperature changes have been shown to affect orthostatic tolerance: heat stress enhances and cold stress reduces orthostatic challenge-induced syncope (Crandall, 2000). In addition, cardiovascular responses during changes in posture are influenced by temperature changes: standing up/working in upright positions for longer periods in hot weather is associated with poor orthostatic tolerance and increased incidence of syncope (Wilson and Crandall, 2011). These investigators have further suggested that alterations in orthostatic tolerance and cerebral blood flow seen in thermal stress are not due to the neural-induced postural reflexes but rather due to changes in cardiac mechanics and function. What effect heat has on orthostatic tolerance is particularly important for workers exposed to hot temperatures while standing. While heat may predispose such workers to collapse and orthostatic intolerance, it could also lay the foundation for proposing countermeasures such as skin cooling that could improve orthostatic tolerance. Except for the Weiss study (Weiss et al., 2006), in which older persons in nursing homes showed greater incidence of orthostatic hypotension in summer, there is no systematic study which has investigated the effects of temperature changes in older persons. More research is needed in this area.

### Orthostatic intolerance in disease conditions

There are many disease conditions that appear to affect both postural and cardiovascular control that might be better explained and treated with the integration of the systems into a single predictive model; these include peripheral neuropathy (e.g., diabetic neuropathy), concussion and long term bedrest itself. Orthostatic intolerance can also arise in patients with stroke (Verma and Eltawansy, 2016), diabetes mellitus (Eguchi et al., 2006), multiple sclerosis (Pintér et al., 2015), Alzheimer's and Parkinson's disease (Bae et al., 2014), and traumatic brain injury (Kanjwal et al., 2010). The use of medications in patients such as the new SGLT-2 inhibitor dapagliflozin could also lead to hypotension and dizziness upon standing up (Chao and Henry, 2010). Similarly, patients with autonomic failure have difficulties elevating vascular resistance (Smit et al., 1999) during standing up, which could predispose them to orthostatic intolerance. While the data from aforementioned studies are from mixed age groups (young adults and middle-aged persons), to what extent these disease conditions affect orthostatic tolerance in older persons needs to be systematically studied.

We are only aware of one study that has investigated in older persons the post-stroke relationship in heart rate variability (HRV; Rodriguez et al., 2017) at rest and during changes in posture. A greater magnitude of decrease in LF HRV modulation upon standing up for the stroke group—indicating a transition to less sympathetic modulation—on average compared to the control subjects was observed. This suggests a paradoxical decrease in sympathetic modulation of the heart rate during orthostatic challenge, which could potentially arise *either* due to ischemic damage affecting nucleus tractus solitarius (NTS) signaling, resulting in constant sympathetic activity, which over time can increase resistance at adrenergic beta-receptors to stimulation *or* there may be a greater inhibition of sympathetic catecholamine release. Patients with hypertension, stroke, and chronic heart disease often display this type of reactivity and the Rodriguez et al. (2017) observation of resting heart rate in the stroke group being lower before and after standing are consistent with either of these theories. While the exact mechanisms for these differences are still unknown, Tang et al. (2012) attributed autonomic dysfunction as a major contributor to the high prevalence of orthostatic hypotension in their stroke patients. Furthermore, the extent of DBP or SBP reduction after standing between the stroke and healthy control groups was not different (Rodriguez et al., 2017). However, the mean BP drop for the stroke group in this study approached the criteria for OH. Earlier studies have reported that a greater risk of syncope occurs when cerebral perfusion reduces in response to a drop in central BP, and that this mechanism is related to a sudden withdrawal of sympathetic activity (Cooke et al., 2004). The greater baseline sympathetic modulation (that is, LF values) in the stroke group could lead to an earlier withdrawal of sympathetic vascular tone, with an increased risk of syncope upon standing up, particularly if beta-adrenergic responses are blunted (Overgaard and Dzavik, 2008).

Another condition that has been shown to increase the susceptibility to orthostatic hypotension is spinal cord injury (SCI; Claydon and Krassioukov, 2006). Indeed, orthostatic maneuvers carried out during mobilization and physiotherapy are associated with hypotension in up to 70% of the patients with SCI (Claydon and Krassioukov, 2006; Popa et al., 2010; Sahota et al., 2012). Although SCI is mostly found in the younger population, older people are at risk of traumatic SCI from falls (Ahn et al., 2015), justifying more studies on the repercussion of SCI on OH in older individuals.

OH that accompanies SCI can also complicate a patient's participation in rehabilitation, especially when carrying out exercises (Claydon and Krassioukov, 2006; Popa et al., 2010). For instance, post-exercise hypotension can occur for up to 12 h following exercise (Claydon et al., 2006). These investigators investigated the effects of graded arm cycling until exhaustion in 29 chronic SCI patients (cervical-level: *n* = 19; thoracic-level: *n* = 8). Following exercise it was observed that MAP decreased in patients with cervical SCI (−9.3 ± 2 mmHg) but increased in thoracic SCI (8.4 ± 5 mmHg; *P* < 0.001). They suggested that these effects might partially arise due to the loss of descending sympathetic nervous control of the heart and vasculature, which accompanies SCI of higher levels. However, these data were obtained from a sample of younger persons. Therefore, interpretations in terms of effects of exercise in older people with SCI must be applied with caution.

## Countermeasures

The search for countermeasures against orthostatic intolerance continues. Even after several decades of investigation, the problem of orthostatic intolerance in older persons remains. As the aging population increases worldwide, there an urgent need to develop innovative countermeasures against orthosatic intolerance. It is important, for example, when intervening in the process in which bedrest confinement leads to orthostatic intolerance and falls, that a holistic multifactorial approach which takes into account key factors such as nutrition, (de)conditioning, muscle loss, cardiovascular and vestibular effects, is followed. Such an approach will lead to more effective management of bedrest confinement, reduced orthostatic intolerance and falls and falls-related injury, and consequently decrease public health costs, costs for older people and their families as well as decrease secondary care dependency.

Outlined below are some of the possible countermeasures that are currently in use or being examined. While not all of these interventions have been tested in older persons, we present here some evidence that these countermeasures can reduce orthostatic intolerance in young and middle aged persons.

### Physical activity/exercise in ambulatory persons and in immobilized persons

Physical exercise is therapeutic for many medical disorders (Jolliffe et al., 2001), and it can also counter muscle wasting in acute illnesses. Muscle wasting during bedrest confinement is highly pronounced at old age (Kortebein et al., 2007), and compromised muscle function is the major obstacle to remobilization in elderly patients (Creditor, 1993). Therefore, physical exercise, applied as early as possible during a hospital stay is now seen as the gold standard in acute hospital care for 75 year + people.

### Aging, bedrest confinement and the need for early remobilization: role of physical exercise

Mobility, especially ambulatory mobility, is an essential aspect of quality of life and independence. As the aging process proceeds, ambulatory ability begins to deteriorate as a result of sarcopenia (aging-associated degenerative loss of skeletal muscle quality, mass and strength; Phillips, 2015) and dynapenia (aging-associated loss of muscle strength not arising due to muscular and/or neurologic diseases; Clark and Manini, 2008). Sarcopenia is a multiple etiological syndrome, thought to partly arise by cell death of α-motoneurons, i.e., the nerve cells that supply muscle fibers with information from the central nervous system. Skeletal muscle fibers, and α-motor neurons of the faster type are likely to be more affected than those of the slow type, which offers an explanation why speed and power are particularly eroded during aging (Vandervoort, 2002; Deschenes, 2004). Muscular atrophy is different from this—it affects slow fibers more than fast fibers, and one best understands it as muscle's self-adaptation to reduced demands (Li et al., 2013).

In addition to aging, illness and injury can impact ambulatory ability by depriving muscles of normal stimulation related to ambulation. Such disuse atrophy combined with sacropenia and dynapenia can result in significant muscle wasting (Kortebein et al., 2007; Suetta et al., 2009) contributing to deconditioning that can have many negative medical consequences (Brown et al., 2004) including risk of falls and falls injury. Almost immediately after hospitalization admission, skeletal muscle deconditioning begins and deficits in activity of daily life (ADL) can be observed as early as day two of hospitalization (Hirsch et al., 1990). If deconditioning severely limits or blocks ambulatory activity then reduction in muscle strength and power can become self-perpetuating. Such muscle deficits are often accompanied by generalized inflammation and metabolic disorders which complicate muscle function recovery. To avoid such a vicious cycle linking bedrest confinement to muscle wasting and further reduced ambulation requires immediate intervention after hospitalization admission to remobilize the patient as early as possible during and after hospitalization (Singh et al., 2008; Goswami, 2017).

When prescribing physical activity as a countermeasure to muscle deconditioning, aspects such as metabolic state of the muscles as well as amount of mechanical stress and myo-electrical activation to be applied must be addressed. Resistance/“strength” training for instance involves large mechanical stress and large myo-electrical activity, and it leads to enlargement mostly of the faster fiber types. Accordingly, resistive training is recommended by many practioners to combat sarcopenia, in the absence of any causative cure. However, resistive exercise regimens that would normally lead to muscle enlargement (hypertrophy) fail to even maintain calf muscle strength and size under bedrest conditions (Alkner and Tesch, 2004; Rittweger et al., 2005)—like many other countermeasures tested against bedrest-induced deconditioning (Pavy-Le Traon et al., 2007). The available literature thus underlines the difficulty and complexity involved in adequately training muscles in bed confined persons. Therefore, one has to be doubtful with regards to the effectiveness of the general care that is currently being carried out in hospitals, as elastic bands or static exercise using gravity as a resistor are bound to be sub-optimal from a muscle physiologist's point of view, while gait and balance training may perhaps help only to improve balance (Mulder et al., 2015).

To be effective, physical interventions need to be not only multidimensional, standardized, and interdisciplinary, but individualized as well. However, such evidence-based intervention programs (let alone individualized) are still lacking for hospitalized older people. As a result, in many hospitals, the acute care of older persons does not incorporate procedures to promote remobilization early enough. Physical exercise interventions are often delayed and the procedures implemented may be based on individual therapist experience leading to non-standardized interventions which are often only applied after substantial loss of muscle mass and function has occurred. Thus patients may be discharged without recovering sufficient physical function leading to a vicious circle of hospital deconditioning leading to injury and further hospitalization and further dependency (Singh et al., 2008; Goswami, 2017).

### Ingestion of water to attenuate OI or postprandial hypotension

The responses to water consumption in young healthy persons are complex with a simultaneous increase in vagal tone, as evidenced by bradycardia and an increase in the high-frequency component of the HRV, and some vasoconstriction, as evidenced by a rise in peripheral resistance (Brown et al., 2005), but no real changes in blood pressure (Jordan et al., 2000; Brown et al., 2005). These changes occur despite an increase in muscle sympathetic nerve activity (MSNA) and higher levels of norepinephrine in plasma. Interestingly, the water-induced vagal stimulation is enhanced by drinking cold water (Girona et al., 2014). In subjects showing some degree of baroreflex impairment, the lack of increased vagal tone may no longer attenuate the vasoconstrictor effects of sympathetic activation and may thus unmask hypertension. Murakami et al. (1993) showed that borderline hypertensives had reduced parasympathetic tone. Parasympathetic withdrawal has been shown to be an important precursor to blood pressure elevations in women with primary hypertension (Dabrowska et al., 1996). Furthermore, a recent paper using HRV (Goit and Ansari, 2016) showed that 120 newly diagnosed hypertensives (37–42 yrs) had lower parasympathetic tone compared to controls. Indeed, in severe autonomic failure patients, water drinking substantially increases blood pressure (Jordan et al., 2000; Cariga and Mathias, 2001) and is associated with increases in plasma norepinephrine (Jordan et al., 2000).

In older persons, the effects of water drinking has been sparsely studied. However, one study has clearly shown that drinking half a liter of water leads to acute increases in blood pressure also in older subjects (Jordan et al., 2000). Therefore, it is no surprise that water ingestion has been proposed to attenuate orthostatic or postprandial hypotension (Shannon et al., 2002). Indeed, drinking half a liter of water in healthy subjects improved orthostatic tolerance (assessed using head-up tilt test followed by graded lower body negative pressure; Schroeder et al., 2002). Intake of approximately half a liter of tap water in <5 min in patients suffering from primary autonomic failure has been shown to improve both the postprandial drop in blood pressure and orthostatic hypotension (Shannon et al., 2002). Similarly, water drinking also decreased the orthostatic tachycardia in patients with idiopathic orthostatic intolerance (Shannon et al., 2002). Grobéty et al. (2015) have also shown recently that ingestion of a light breakfast of about 400 Kcal in older people (67 ± 1 years) lead to reductions in both diastolic and systolic BP, decreases that were not found in younger control subjects (25 ± 1 years), and that the prior ingestion of 500 mL water cut by half the decrease in systolic BP.

### Mental challenge as a countermeasure

#### Effects of mental challenge

Mental challenge increases heart rate, cardiac output, force of cardiac contraction, and blood pressure (Lackner et al., 2010). It is accompanied by venoconstriction and arterial vasoconstriction in the splanchnic, renal, and cutaneous circulations (Callister et al., 1992). The mental arithmetic task has also been shown to elevate epinephrine, norepinephrine and plasma renin activity in venous blood.

#### Beneficial role of mental challenge as a potential countermeasure against orthostatic intolerance

In a pilot study, Goswami et al. (2012a) have shown that the orthostatic tolerance of young persons can be improved by the application of mental arithmetic. Mental arithmetic (MA) done during head up tilt (HUT) leads to larger increases in heart rate—which can maintain cardiac output—than heart rate changes caused by MA or HUT alone (Lackner et al., 2010).

Mental challenge-induced increases in sympathetic activity also affect the venous system. As 70% of total blood volume is approximately in the venous system, and veins are strongly controlled by the sympathetic system (reviewed in Pang, 2001), the venous system can influence the amount of blood returning to the heart—and consequently the cardiac preload—when its capacity is changed. As discussed above, mental challenge-increases the sympathetic activity, which in turn increases cardiac output; this could be sustained by larger increases in venous tone, greater mean circulatory filling pressure and thus greater venous return (see Guyton's analysis, Montani and Van Vliet, 2009). The results of Goswami et al. (2012a) are supported by studies which have reported that mental challenge is accompanied by splanchnic vasoconstriction (Callister et al., 1992).

The responses to mental challenge conducted in upright position are unsurprising, as mental challenge induced central drive modulates physiological responses (e.g., cardiovascular reflexes, Ross and Steptoe, 1980). For instance, mental loading can affect baroreflex function via it's actions at the hypothalamus, pons or medulla (Stephenson, 1984). It also appears that the application of mental stress modifies the input to the baroreceptors, which occurs during orthostatic loading (Goldstein and Shapiro, 1988).

#### Using mental stressors: advantages of mental arithmetic

Mental arithmetic, Stroop color-word conflict, and public speaking are commonly used mental stressors (Steptoe and Vogele, 1991). Mental arithmetic is a calculations task that involves several cognitive mechanisms, including a consciously executed calculation method or a direct retrieval of the answer from memory (McCloskey et al., 1985). Overall, the frontal lobe is involved in calculations that are conscious but temporo-centro-parietal activity is involved in direct automatic retrieval of results (Pauli et al., 1996). Mental arithmetic can be performed in all circumstances, including emergency ones (that is, at the bedside or roadside). However, caution must be observed when extrapolating the effects of mental arithmetic on orthostatic intolerance in older persons, as the data from Goswami et al. (2012a) were obtained mainly from younger subjects.

#### Timing of mental challenge application

Goswami et al. (2012a) propose that mental challenge could be used as a countermeasure to prevent orthostatic intolerance, particularly in persons who feel dizzy upon standing up, or to counteract hypotension that often occurs during hemodialysis. However, as the cardiovascular responses to mental arithmetic decrease over time, an important aspect that must be considered when using mental challenge as a countermeasure is the *exact timing of the application*. Lackner et al. (2010) have reported that a reduction in the hemodynamic responses to orthostatic and mental challenges occurs over time when each of these stressors is applied alone (Lackner et al., 2010). For instance, mental challenge induced cardiovascular effects occur maximally within the first two to three min of application (Lackner et al., 2010). As longer applications of mental challenge can lead to habituation/adaptation of responses (thus decreasing the effectiveness of mental challenge), Goswami et al. (2011) recommended that mental arithmetic should be applied only 2–3 min preceding the occurrence of orthostatic intolerance (i.e., if a person is known to become dizzy upon standing up, they should start their mental arithmetic calculations 2–3 min prior to standing up).

### Other potential countermeasures in older persons

Some other countermeasures that could potentially prevent orthostatic intolerance include standing up slowly (de Bruïne et al., 2017). *Standing up slowly* in older persons with histories of orthostatic hypotension has been shown to antagonize the blood pressure decreases within the first 15 sec of changes in posture/ standing (de Bruïne et al., 2017).

Recent evidence also suggests that cognitive training (Goswami et al., 2015) and nutritional supplementation (Muscaritoli et al., 2017)—with and without physical activity- affect vascular function and, therefore, could potentially improve orthostatic intolerance. Aspects of these interventions are now examined.

#### Cognitive training

The concept of using cognitive training as a countermeasure in preventing functional decline originates from the observations that cognitive interventions promote functional outcomes, particularly improvements in mobility in older sedentary persons (Verghese et al., 2010). With a novel computerized cognitive training (CCT) intervention, developed from an underlying brain-based model, Marusic et al. (2014) and Goswami et al. (2015) recently assessed whether CCT (provided as simulation of walking through a labyrinth) could improve cognitive functioning—and at the same time mitigate possible bedrest-associated decline from a functional perspective. The bedrest confinement, carried out in healthy persons aged between 18–30 yrs (younger persons) and 55–65 yrs (middle aged to older persons), lasted up to 14 days, during which the participants were not allowed to leave the bed. While bedrest triggered various functional and metabolic adaptations in older and younger individuals (Pisot et al., 2016; Soavi et al., 2016), it had no remarkable effects on cognition (Dolenc and Petric, 2013)—which is in agreement with some of the previous bedrest studies (for review see Lipnicki and Gunga, 2009; Marusic et al., 2014). Indeed, CCT intervention during bedrest confinement was effective in enhancing cognitive functioning at the end of bedrest and had sustained/long-term positive effect on cognition (Marusic et al., 2016). Improved cognitive functioning was shown to be further effective in positively affecting functional performance parameters (e.g., improved dual-task walking condition and reduced gait variability, Marusic et al., 2015), which could potentially reduce number of falls following prolonged bedrest, especially in older persons. Recently, a neuroprotective mechanism of CCT has been proposed by Passaro et al. (2017), showing as an unaltered plasma brain-derived neurotrophic factor (BDNF) only in the CCT group during bedrest. The control group of older adults (who did not do any CCT during bedrest), on the other hand, showed a significant increase in BDNF level at the end of bedrest (Passaro et al., 2017), which was further interpreted as protective overshooting of the brain to counteract bedrest-related negative effects (Soavi et al., 2016; Passaro et al., 2017). Additionally, CCT provided during bedrest confinement also led to a prevention of decreases in vascular function changes in the older persons group during the bedrest confinement (Goswami et al., 2015). As the changes in vascular function and/vascular reactivity have been shown to contribute to orthostatic intolerance following bedrest confinement as well as to cardiovascular diseases Goswami et al. (2015) propose that cognitive challenge during bedrest confinement may prevent bedrest-induced pathological effects on the vasculature. A limitation of the Goswami et al. (2015) study was that, due to ethical constraints, much older/elderly persons could not be included into the study. However, the pilot data generated from the bedrest study can be used as a basis for motivating the ethics committees to allow recruitment of persons with higher ages than the one that were used in the Goswami et al. (2015) study.

Finally, as mental challenge causes increased blood flow in skeletal muscles (Kuipers et al., 2008), and in addition cognitive training (emphasizing virtual movement) could have effects on brain functions related to walking and mobility (thus potentially affecting peripheral blood flow), further research is needed to assess whether cognitive training during bedrest confinement could prevent post-bedrest induced orthostatic intolerance in older adults.

#### Nutritional therapy

Nutritional therapy, along with resistance training affects muscle mass in healthy older adults (Strandberg et al., 1985). Specifically, a protein-enriched diet, approximately equal to 1.3 g · kg^−1^ · d^−1^ provided by red lean meat has been shown to improve the effects of progressive resistant training on lean tissue mass and muscle strength in older women (Daly et al., 2014). However, a Cochrane review has recently concluded that nutritional therapy can reduce healthcare costs but overall the evidence from the studies is too heterogeneous and of limited quality for concluding whether malnutrition or its treatment helps in reducing re-admissions (Muscaritoli et al., 2017).

#### Respiratory training

Inspiratory-expiratory pressure threshold respiratory training has recently been shown to reduce the incidence of orthostatic hypotension in more than 50% of patients with SCI (Aslan et al., 2016; Legg Ditterline et al., in press). Respiratory training induced increases in respiratory capacity leads to increased sympathetic activation and baroreflex effectiveness as well as improvements in respiratory-cardiovascular interactions during central hypovolemia induced by changes in posture (sit to stand) in patients with SCI. However, the potential benefit of respiratory training in older patients suffering from OH has never been tested.

## Summary

This review outlines orthostatic intolerance connected to age, bedrest confinement, and diseases. Aging-associated illness or injury due to falls often leads to hospitalization. As older patients spend significant amounts of time during hospital admission lying in bed, the consequences of bedrest confinement such as physiological deconditioning, functional decline, and orthostatic intolerance represent a central challenge in the care of the vulnerable older population.

The review also discusses contributing mechanisms of orthostatic intolerance and examines countermeasures such as exercise, water drinking, and mental arithmetic. The *timing* of the countermeasure application is also considered. Finally, this paper emphasizes the importance of an active life style in old age and why early re-mobilization following bedrest confinement is crucial in preventing orthostatic intolerance, falls and falls-related injuries in older persons.

## Clinical implications and future directions

There is a need to integrate cerebrovascular and cardiovascular variables into a combined analysis to allow for the development of improved orthostatic intolerance and fall risk-screening monitoring in older persons. Hand held devices for the analysis of global HRV (a key aspect for monitoring autonomic function (Grote et al., 2013; Osborne et al., 2014; Patel et al., 2016; Moser et al., 2017; Rodriguez et al., 2017) for instance, are currently available, which helps in streamlining data collection at homes or in mobile settings. Assessing the autonomic system using a simple method that does not utilize the baroreceptor reflex (e.g., oculocardiac reflex) could also help in separating vessel disease and central autonomic dysfunction at the bedside (McLaren et al., 2005). Assessment of HRV and blood pressure variability is also important as HRV has been associated with mortality following myocardial infarction while greater blood pressure variability is associated with greater disability following stroke.

Additionally, rehabilitation post-stroke could benefit from combination of mobile monitoring of the autonomic and cardiovascular systems with effective physical therapy interventions to ensure that older persons are protected from muscle wasting and muscle loss after stroke. Future sit-to-stand protocols that include MSNA evaluation, by which the magnitude and timing of sympathetic responses could be measured, could be carried out to clarify the relationship between MSNA and HRV. All these aspects are important in the asssement of orthostatic intolerance risk in patients during hospitalization and upon discharge.

Finally, there is a need to implement more integrated screening procedures during hospital admission to personalize countermeasures against orthostatic intolerance, which can arise due to bedrest confinement.

## Author contributions

NG conceived the idea and wrote the manuscript. AB supported in editing the manuscript and contributed to sections related to cardio-postural interactions. HH-S helped in writing the manuscript. J-PM supported in editing the manuscript and contributed to sections related to water drinking as well as post-prandial hypotension.

### Conflict of interest statement

The section related to mental stress is based on the Doctoral work of NG. The other authors declare that the research was conducted in the absence of any commercial or financial relationships that could be construed as a potential conflict of interest.

## Abbreviations

ADL: Activity of daily life
BP: Blood pressure
BP-MCA: Blood pressure corrected to the level of middle cerebral artery
CCT: Computerized cognitive training
CO: Cardiac output
CVRi: Cerebrovascular resistance index
DBP: Diastolic blood pressure
ETCO2: End tidal CO_2_ pressure
HF: High frequency
HR: Heart rate
HUT: Head up tilt
JNC: Joint National Committee
LF: Low frequency
MA: Mental arithmetic
MA + HUT: Mental arithmetic in HUT subjects
MAP: Mean arterial (blood) pressure
MFV: Mean flow velocity (Cerebral artery)
MSNA: Muscle sympathetic nerve activity
NTS: Nucleus tractus solitaries
OH: Orthostatic hypotension
OI: Orthostatic intolerance
PP: Pulse pressure
SBP: Systolic blood pressure
SCI: Spinal cord injury
SGLT-2: Sodium glucose co-transporter 2
SV: Stroke volume
TPR: Total peripheral resistance.
